# Feasibility of the aktivplan Digital Health Intervention for Regular Physical Activity Following Phase II Rehabilitation: Protocol for a Mixed Method Randomized Controlled Pilot Study (ACTIVE-CaRe Pilot)

**DOI:** 10.2196/73704

**Published:** 2025-09-15

**Authors:** Dirk Leysen, Bernhard Reich, Anna Eleonora Carrozzo, Rik Crutzen, Vincent Grote, Devender Kumar, Barbara Mayr, Josef Niebauer, Franziska Pfannerstill, Eva Maria Propst, Daniela Wurhofer, Mahdi Sareban, Jan David Smeddinck, Gunnar Treff, Stefan Tino Kulnik

**Affiliations:** 1 Ludwig Boltzmann Institute for Digital Health and Prevention Salzburg Austria; 2 Department of Health Promotion, Care and Public Health Research Institute, Maastricht University Maastricht The Netherlands; 3 Salzburg Research Forschungsgesellschaft Salzburg Austria; 4 REHA Zentrum Salzburg Salzburg Austria; 5 University Institute of Sports Medicine, Prevention and Rehabilitation Salzburg Austria; 6 Institute of Molecular Sports and Rehabilitation Medicine, Paracelsus Medical University Salzburg Austria; 7 Ludwig Boltzmann Institute for Rehabilitation Research Vienna Austria

**Keywords:** app, behavior change, cardiac rehabilitation, cardiovascular disease, digital technology, eHealth, exercise, habit formation, mHealth, mobile health, secondary prevention

## Abstract

**Background:**

Patients with cardiovascular disease (CVD) often encounter challenges in establishing and maintaining heart-healthy physical activity habits, even after successfully completing a cardiac rehabilitation program. Digital health technologies hold promise to support long-term habit formation in the secondary prevention of CVD. The aktivplan digital health intervention has been developed to support patients with CVD in establishing long-term heart-healthy physical activity habits.

**Objective:**

The primary study aim is to pilot and assess the feasibility of a future randomized controlled trial design to investigate the effectiveness of the aktivplan intervention and to assess the usability, user experience, and acceptance of the aktivplan app. The secondary objective is to collect clinical and safety outcomes.

**Methods:**

This multicenter, mixed method, randomized controlled pilot study aims to recruit 40 patients with an established diagnosis of CVD or with increased risk of CVD (physically inactive along with 1 further CVD risk factor) who are undergoing phase II rehabilitation at 2 rehabilitation centers in Austria. Participants will be allocated to the intervention or standard care control group by stratified randomization and will be monitored for 10 weeks after discharge from phase II rehabilitation. Participants, health care professionals, and outcome assessors are not masked (blinded) to group allocation. Data collection will include recruitment and drop-out rate; data completeness; adherence to the intervention; usability, user experience, and user acceptance questionnaires; technical reliability of the intervention; clinical assessments (exercise capacity, physical activity behavior, and CVD risk factors); adverse events; self-reported outcome measures (health-related quality of life, exercise self-efficacy, depression and anxiety, and kinesiophobia); patient interviews, and focus groups with health care professionals. Quantitative data will be analyzed descriptively, and 95% CIs will be calculated for recruitment and drop-out rates and for data completeness. No confirmatory inferential statistical analysis or hypothesis testing will be conducted. Qualitative data will be analyzed thematically by framework analysis.

**Results:**

A total of 34 participants were recruited between October 2023 and May 2024. Data collection was completed in August 2024. Currently, the data are being analyzed and prepared for publication. The first publication of feasibility results is expected by summer 2025.

**Conclusions:**

This pilot study is expected to generate valuable and comprehensive insights to inform the study design of a future definitive effectiveness trial of the aktivplan intervention, guide the need for further iteration of the aktivplan app before entering a definitive trial, and inform future implementation strategies for the intervention.

**Trial Registration:**

ClinicalTrials.gov NCT06025526; https://clinicaltrials.gov/study/NCT06025526

**International Registered Report Identifier (IRRID):**

DERR1-10.2196/73704

## Introduction

### Background

Cardiovascular disease (CVD) remains the most common cause of death worldwide, with 18.6 million deaths attributed to CVD in 2019 [1]. In Austria, CVD was the most common cause of death in 2022 (34.3% of all deaths) and the most frequent discharge diagnosis for all acute hospital admissions (11.6%) [2]. Cardiac rehabilitation constitutes an important care pathway for the secondary prevention of CVD [3,4]. Cardiac rehabilitation programs aim to support patients in implementing a lifelong heart-healthy lifestyle, including regular physical activity, for example, through joining cardiac exercise groups or through self-directed training at home [3]. As a general recommendation, patients should carry out aerobic physical activity at a moderate or moderate-to-high intensity at least 3 times per week, and ideally 6 to 7 times per week. Aerobic physical activity should amount to at least 150 minutes per week at moderate intensity, or 75 minutes per week at vigorous intensity, or an equivalent combination thereof. In addition, patients should perform muscle-strengthening exercises for the large muscle groups twice per week with 8 to 15 repetitions per set. The overall training volume should amount to an energy expenditure of 1000 to 2000 kcal per week [5-7].

High-level scientific evidence demonstrates the effects of cardiac rehabilitation programs on reducing long-term morbidity and mortality. Cardiac rehabilitation has therefore received the highest class of recommendation in clinical guidelines for patients with chronic heart failure, chronic cardiovascular risk, after myocardial infarction with ST-elevation, after myocardial revascularization, and for CVD prevention in clinical practice [6]. The cardiac rehabilitation pathway is organized according to phases, whereby phase I describes the period of acute hospital admission. Phase II describes a structured and supervised program, delivered in an inpatient or outpatient setting and lasting several weeks. It aims to provide patients with the knowledge and awareness of the health-promoting effects of regular physical activity and other heart-healthy lifestyle choices, and to impart the skills to effectively plan and implement these CVD preventive behaviors [5,6].

However, it is a well-documented problem that many patients with CVD struggle to establish regular physical activity habits. In the EUROASPIRE V survey of 8261 patients with coronary artery disease in 27 European countries, only 34% of respondents reported performing regular physical activity (ie, ≥30 minutes on average 5 times a week) [8].

The same is true for people with an increased risk of CVD who have not yet developed the disease (ie, primary prevention of CVD) [9]. According to the Austrian Health Interview Survey, only 48.1% of men and 45.1% of women aged between 18 and 64 years meet the World Health Organization (WHO) recommendation to carry out at least 150 minutes per week of endurance-type physical activity at moderate intensity [10].

Moreover, successful completion of a phase II cardiac rehabilitation program does not necessarily lead to establishing long-term heart-healthy physical activity habits. The systematic review by ter Hoeve et al [11] included 26 randomized controlled trials and reported limited and conflicting evidence that cardiac rehabilitation programs improved long-term (≥6 months) maintenance of physical activity recommendations compared to groups who did not attend cardiac rehabilitation. These findings are corroborated in the systematic review by Dibben et al [12] who included 40 randomized controlled trials and found that ≤12 and >12 months after a cardiac rehabilitation program, only 26% of physical activity outcome comparisons showed statistically significant differences in favor of the cardiac rehabilitation groups.

This urgent and challenging problem has also been highlighted in statements and position papers from medical and scientific societies. For instance, the European Research Area Network for Cardiovascular Diseases has defined this problem as a priority topic for scientific research in 2019 [13]:

Maintaining a healthy lifestyle appears to be difficult for an increasing majority of the population, despite the general acknowledgment of the benefit of keeping risk factors for cardiovascular disease as low as possible. This not only applies to young people—for whom cardiovascular disease will often not develop for some time—but also to patients who, after a period of intensive rehabilitation, have difficulties maintaining the healthy lifestyle by themselves. So far, research has not delivered any effective strategy to improve healthy lifestyle maintenance. Research in this area will have to explore whether optimal use of new technologies like intelligent wearables will offer personalized, innovative, and effective solutions to this problem.

The current European Society of Cardiology guideline on CVD prevention mirrors this statement, describing a specific evidence gap concerning physical activity “Implementation of strategies to achieve long-term adherence to PA [physical activity]” and a further evidence gap that suggests a possible solution through digital technologies: “Evaluation of the effects of eHealth tools in promoting PA [physical activity]” [14].

Digital technologies offer potential innovative solutions for long-term and sustainable habit formation for independent, regular, heart-healthy exercise. The systematic review of Wongvibulsin et al [15] included 31 studies to analyze the potential of digital health technologies in cardiac rehabilitation. The most used technologies were smartphones or mobile devices (20/31, 65%), websites or web-based portals (18/31, 58%), and email or SMS text message communications (11/31, 35%). The authors concluded that digital technologies have the potential to enhance care and broaden access to cardiac rehabilitation through tailored interactive interventions. Furthermore, the reviews by Meinhart et al [16] and Luijk et al [17] indicate that supporting physical activity through digital technologies following a cardiac rehabilitation program can achieve higher physical activity and physical performance outcomes compared to standard care (without digital technology support following a cardiac rehabilitation program).

Digital health interventions typically comprise of 2 interrelated components: the digital technology and the behavioral strategies implemented through and alongside the technology. These behavioral strategies are often based on established behavior change techniques (BCTs), such as goal setting, self-monitoring, feedback on performance, and education. However, disentangling the respective contributions of the digital technology and the underlying BCTs remains challenging. For example, in the review by Meinhart et al [16], 5 distinct BCTs were incorporated in almost all study interventions, making it difficult to distinguish their relative importance: goal setting (13/13, 100%), self-monitoring (13/13, 100%), feedback on exercise (13/13, 100%), physician or expert involvement (12/13, 92%), and tailored exercise prescription (11/13, 85%). The systematic review by Aguiar et al [18] described the most frequently implemented BCTs in 24 studies of mobile health interventions for patient adherence: feedback and monitoring (20/24, 83%), goals and planning (14/24, 58%), associations (14/24, 58%), shaping knowledge (12/24, 50%), and personalization (7/24, 29%). While there was mixed evidence of effectiveness, the BCTs that were most represented in positive studies were feedback and monitoring, goals and planning, associations, and personalization. Overall, this body of evidence illustrates the potential of digital interventions for improving patient outcomes, but it also highlights the heterogeneity and complexity of digital interventions, necessitating detailed and transparent descriptions of both their technological components and behavioral strategies [19].

### This Study

This study aims to investigate the feasibility of a future randomized clinical trial on a novel digital health intervention, aktivplan [20]. aktivplan has been developed specifically to support patients in establishing regular heart-healthy physical activity habits following completion of a phase II rehabilitation program in the Austrian health care context. In addition to feasibility, this study will also evaluate the usability and acceptability of the aktivplan app.

## Methods

### Aim

The primary aim of this study is to pilot and assess the feasibility of a future randomized controlled trial design that will investigate the effectiveness of the aktivplan digital health intervention. In addition, the study will evaluate the usability, user experience, and acceptance of the aktivplan app, providing information on potential improvements.

The secondary aim of this study is to collect clinical outcomes to inform the sample size calculation for a future definitive effectiveness trial and to demonstrate the patient safety of the intervention.

### Study Design

This is a multicenter, mixed method, randomized controlled pilot study. Forty patients undergoing phase II rehabilitation will be recruited from 2 study sites (20 patients per site). Study participants will be individually randomized to the intervention group (aktivplan digital health intervention) or control (standard care, ie, not including any routinely provided digital technologies) and will be followed up for 10 weeks after discharge from phase II rehabilitation.

The reporting of this study protocol adheres to the SPIRIT (Standard Protocol Items: Recommendations for Interventional Trials) statement [21], the CONSORT (Consolidated Standards of Reporting Trials) statement extension to randomized pilot and feasibility trials [22], and the TIDieR (Template for Intervention Description and Replication) checklist [23]. The SPIRIT, CONSORT, and TIDieR checklists are provided as Multimedia Appendices 1-3.

### Ethical Considerations

This study protocol was reviewed and received favorable ethical opinion from the research ethics committees of the Austrian federal states of Salzburg (1065/2023) and Vorarlberg (EK-2-14/2023). Participation in this study is voluntary and all participants give written informed consent to take part in the study. Participants retain the right to withdraw from the study at any time and without giving a reason. In accordance with applicable medical device and product regulations in Austria, the study was registered with the Austrian Federal Office for Safety in Health Care (102214904). For general transparency and disclosure, the study was prospectively registered on ClinicalTrials.gov (NCT06025526). No person-identifying data or images will be published in the dissemination of this research.

### Regulatory Considerations

This study protocol is in accord with national and international ethical and regulatory standards, including the Declaration of Helsinki and the International Council for Harmonization of Technical Requirements for Pharmaceuticals for Human Use Guideline for Good Clinical Practice [24], the European Union Medical Device Regulation [25], and the European Union General Data Protection Regulation [26].

### Indemnity Insurance and Monitoring Visits

Study participants, site principal investigators, and other clinical personnel at the study sites are covered by indemnity through a study insurance. Data monitoring is performed by the study steering committee in periodic meetings and by independent monitoring visits at the study sites.

### Setting

The study is set in 2 rehabilitation centers in Austria, 1 outpatient rehabilitation center in the city of Salzburg (study site 1), and 1 inpatient rehabilitation clinic in the rural Montafon valley (study site 2). The outpatient rehabilitation center offers phase II rehabilitation of 4- to 6-week duration for adults with cardiac, pulmonary (including post–COVID-19 condition), metabolic, oncological, and orthopedic conditions. The inpatient rehabilitation center provides phase II rehabilitation stays of 3- to 4-week duration for adults with cardiac, neurological, orthopedic, and psychiatric conditions.

### Participants

All patients admitted to phase II rehabilitation at study site 1 and all patients admitted to phase II cardiac rehabilitation at study site 2 will be screened against predefined eligibility criteria. The inclusion and exclusion criteria are presented in Textbox 1.

**Inclusion and exclusion criteria.**

**Inclusion criteria**

- Adult patients who are enrolled either in a phase II cardiac rehabilitation program or who are enrolled in phase II rehabilitation due to a noncardiac indication but who present with increased cardiovascular risk (ie, physical inactivity plus at least 1 of the following cardiovascular disease risk factors: smoking, hyperlipidemia, arterial hypertension, diabetes, or obesity).
- Possess and use a smartphone compatible with the aktivplan digital health intervention (Android [version 4.4; Google LLC] or Apple iOS [version 11.0; Apple Inc] or above and internet access).

**Exclusion criteria**

- Existent use of a digital intervention to support regular heart-healthy physical activity by the patient already established in standard care at the time of recruitment.
- Medical contraindications to: symptom-limited incremental cycle ergometry, regular heart-healthy physical activity, exercise and sports, or the use of a smartphone.
- Insufficient cardiovascular exercise capacity (eg, due to severe impairment of the musculoskeletal system).
- Participation of the patient in another clinical trial within the last 6 months.
- Lack of mental capacity to consent to study participation (addiction or other illnesses that do not allow the person to assess the nature, scope, and possible consequences of participation in the study).
- Pregnancy in the third trimester, or pregnancy in the first and second trimester with a medical contraindication to physical activity, exercise, and sports.
- Breastfeeding women with a medical contraindication to physical activity, exercise, and sports.
- Indications that the patient is unlikely to comply with the protocol (eg, unable to commit to attending follow-up appointments).

### Sample Size

The sample size of 40 patients (20 patients per site) reflects the typical sample size for a clinical pilot study [27] and allows the detection of problems (eg, participant withdrawals) with a prevalence of 7.5% with 95% confidence [28]. This sample size is not powered for statistical hypothesis testing of intervention effectiveness because this is not the purpose of this study.

### Recruitment

All patients admitted to phase II rehabilitation at study site 1 and all patients admitted to phase II cardiac rehabilitation at study site 2 will be screened against the inclusion and exclusion criteria by the site principal investigators. The eligibility of each individual patient will be documented in a screening log, including the reasons for exclusion.

All patients who meet the eligibility criteria will be invited to take part in the study by the site principal investigators. The site principal investigators explain the study, provide the study information sheet, and answer any questions about the study. Patients will be given at least 48 hours to consider participation in the study. Patients who express interest in participating in the study will be asked to provide written informed consent, which is required before study initiation.

### Randomization

Participants will be individually randomized to the intervention or control group. Randomization will be stratified by study site, participant sex, and participant exercise capacity (peak power <1.3 Watts/kg, 1.3-1.8 Watts/kg, and >1.8 Watts/kg in incremental cycle ergometry). The randomization schedule will be prepared in advance by the study statistician who is not involved in participant recruitment. The *blockrand* package of the statistical program R (R Foundation for Statistical Computing) will be used [29]. The package enables the randomized allocation of participants for clinical and experimental studies in which participants are recruited individually and sequentially. The randomization schedule will be stored securely at the study sponsor’s office (Ludwig Boltzmann Institute for Digital Health and Prevention), which is at a different location to the study sites. Random allocation is concealed, as personnel at the study sites have no access to the randomization schedule. After a new study participant has been recruited, the site principal investigator informs the study sponsor of the patient’s sex and exercise capacity, and the randomization result is then returned to the site principal investigator.

### Description of the Intervention

The aktivplan digital health intervention was developed at the Ludwig Boltzmann Institute for Digital Health and Prevention, Austria, in a human-centered process with both co-design and iterative participatory design elements, involving health care professionals and patients with cardiac problems between 2020 and 2022 [30]. The core feature of the aktivplan app is a digital calendar for planning regular heart-healthy physical activity. Health care professionals access this calendar via a website or web app (view for health care professionals), and patients access the calendar via an app on their smartphone, tablet, or—if required—via a website on a smartphone, tablet, or computer (view for patients). Before discharge from the phase II rehabilitation program, the aktivplan app is introduced to the patient in a 1:1 consultation with a health care professional experienced in exercise prescription (eg, physiotherapist, sports scientist, and exercise physiologist). This consultation is conducted according to the principles of shared decision-making [31]. A personalized heart-healthy physical activity plan is agreed upon, which the patient should carry out independently following discharge from the phase II rehabilitation program. The health care professional enters this training plan into the aktivplan calendar. Using the aktivplan app, the patient can view their planned physical activity sessions, mark sessions as completed, or enter additional unplanned physical activities (eg, spontaneous activities that are self-initiated by the patient). The health care professional can view the calendars and assess adherence with personal physical activity plans for all patients under their care.

In addition to this core functionality, the aktivplan digital health intervention includes further distinct features, such as automated messages to the patient with motivational content or reminders of planned physical activity sessions, personalized goal setting, and the option for the health care professional to give the patient feedback and contact them via an in-app message. Further details on the aktivplan digital health intervention are given in the TIDieR checklist in Multimedia Appendix 3. Details of the logic model and psychological and behavior change theory underpinning the aktivplan digital health intervention have been published previously [19].

### Description of the Comparator

The control group will receive usual care according to Austrian national standards, which does not include any digital interventions or digital technology provision during or following phase II rehabilitation. No placebo or comparative treatment will be offered. In addition to standard care, patients in both study groups may take individual action to support their adherence to physical activity recommendations (eg, join an exercise class or gym, hire a personal trainer, and download a freely available physical activity app), and any such activities will be documented by self-report.

After completing their participation in the study, participants who are randomized to the control group will be offered the aktivplan physical activity planning session and use of the aktivplan app for a period of 2 weeks. This offer will be made following completion of study participation, and no study data collection will be conducted during this period. This offer is made for ethical reasons and to counteract possible premature withdrawal from the control group.

### Procedure

**Figure 1:**
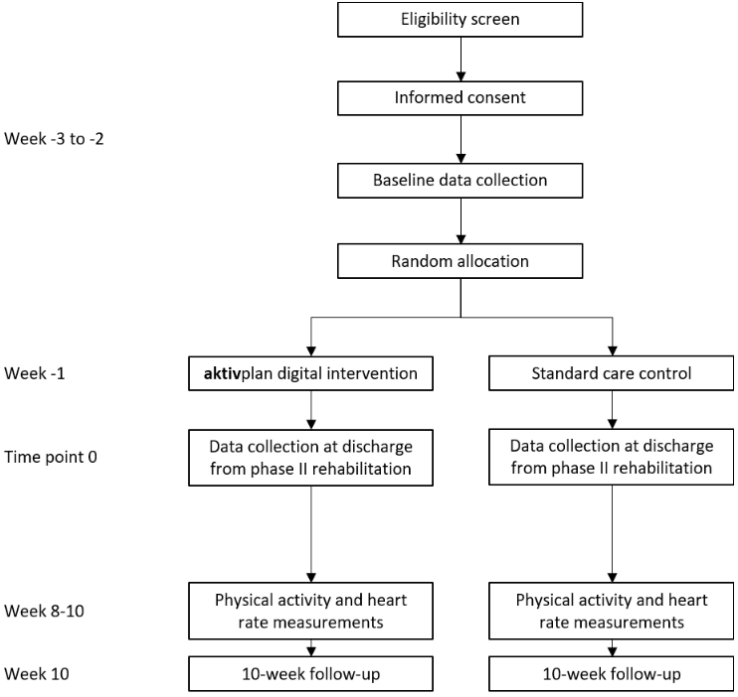
CONSORT flow diagram.

**Figure 2:**
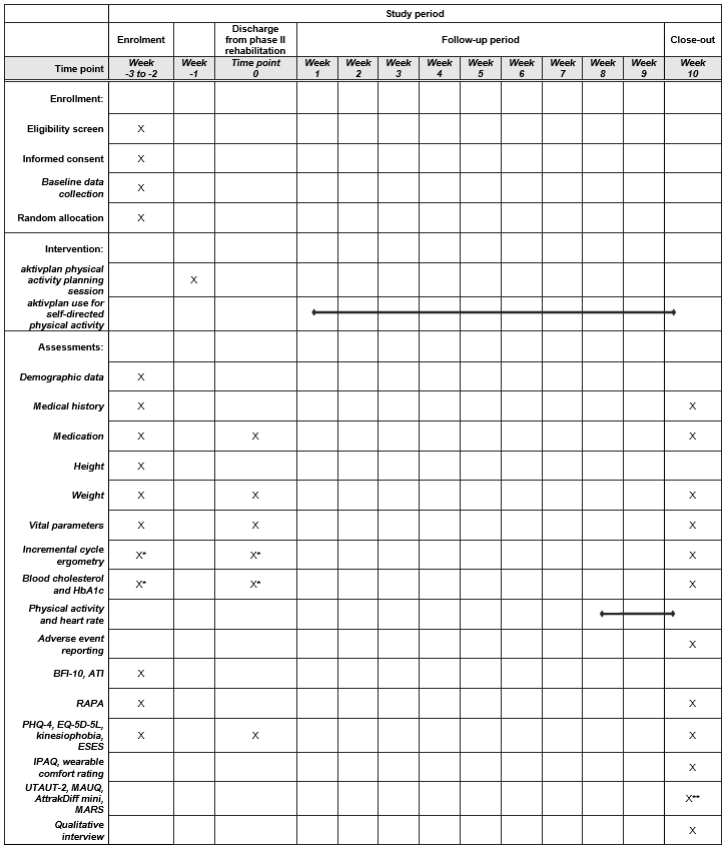
Schedule of enrollment, interventions, and assessments according to the SPIRIT (Standard Protocol Items: Recommendations for Interventional Trials) statement. *collected from routine clinical data, **for participants in the intervention group only. ATI: Affinity for Technology Interaction; BFI-10: Big Five Inventory; ESES: Exercise Self-Efficacy Scale; HbA1c: glycated hemoglobin; IPAQ: International Physical Activity Questionnaire; MARS-G: Mobile App Rating Scale; MAUQ: mHealth App Usability Questionnaire; PHQ-4: Patient Health Questionnaire 4; RAPA: Rapid Assessment of Physical Activity; UTAUT-2: Unified Theory of Acceptance and Use of Technology 2.

The CONSORT flow diagram is presented in Figure 1. The SPIRIT checklist is presented in Figure 2. Study procedures are presented in Textbox 2.

**Study procedures.**

1. Screening for eligibility: all patients admitted to phase II rehabilitation programs at the study sites are screened against study eligibility criteria.
2. Recruitment: all eligible patients are invited to take part in the study. Consent is obtained from those who express interest to participate.
3. Baseline data collection (weeks −3 to −2): baseline data are collected. A detailed description of measures is provided subsequently and in Figure 2.
4. Randomization: patients are randomly allocated to the intervention or control group. Random allocation is concealed and stratified according to study site, patient sex, and patient exercise capacity.
5. aktivplan physical activity planning session (week −1): participants allocated to the intervention group receive the aktivplan physical activity planning session before discharge from the phase II rehabilitation program.
6. Data collection at discharge from phase II rehabilitation (time point 0): data are collected at discharge from phase II rehabilitation. A detailed description of measures is provided subsequently and in Figure 2.
7. Follow-up period: patients in both study groups are followed up for 10 weeks after discharge from phase II rehabilitation. Patients in the intervention group use the aktivplan digital health intervention.
8. Physical activity and heart rate measurements (week 8-10): patients in both study groups receive wearable sensor devices (accelerometer and heart rate sensor) for continuous measurement during waking hours for 3 weeks (weeks 8-10 of the follow-up period). A detailed description of measurements is provided subsequently.
9. 10-week follow-up (week 10): Data are collected in the concluding study visit. A detailed description of measures is provided subsequently and in Figure 2.
10. aktivplan digital intervention for patients in the control group: Following completion of the study, patients in the control group are offered to receive the aktivplan physical activity planning session and use the aktivplan app for 2 weeks. No study data collection will be conducted during this period.

### Data Collection From Participants

#### Data From Routinely Conducted Assessments and Clinical Records

Demographic data and height will be recorded at week –3 to –2. Blood tests (cholesterol and glycated hemoglobin), medication, body mass, vital parameters, and incremental cycle ergometry will be recorded 3 times (at week –3 to –2, time point 0, and week 10). Adverse event reporting will take place at week 10.

#### Self-Reported Questionnaires

At week –3 to –2, time point 0, and week 10, study participants in the intervention and control groups will complete questionnaires on kinesiophobia (selected items from the Tampa Scale of Kinesiophobia [32]), exercise self-efficacy (Exercise Self-Efficacy Scale [33]), depression and anxiety (Patient Health Questionnaire-4 [34]), and health-related quality of life (EQ-5D-5L [35]). At week −3 to −2, they will complete a personality questionnaire (Big Five Inventory [36]) and the Affinity for Technology Interaction questionnaire [37]. At week −3 to −2 and at week 10, they will complete the Rapid Assessment of Physical Activity questionnaire [38]. At week 10, they will complete the International Physical Activity Questionnaire [39] and a questionnaire rating the comfort of the wearable sensor devices (Comfort Rating Scale [40]).

At week 10, study participants in the intervention group will complete questionnaires on the usability and user experience of the aktivplan app, including the mHealth App Usability Questionnaire [41], the Unified Theory of Acceptance and Use of Technology 2 questionnaire [42], the Mobile App Rating Scale [43], and the AttrakDiff mini questionnaire [44]. For some of these questionnaires, only selected relevant sections are used.

#### aktivplan Use Tracking

During the 10-week intervention period, use of the aktivplan app will be automatically tracked, including participants’ log-in and duration, planned physical activity sessions that have been marked as completed by participants, and additional physical activity sessions that participants enter into the app. Adherence will be quantified as a percentage of planned physical activity sessions that have been marked as completed by participants. Engagement with the app will be quantified by the time spent interacting with the app. Notably, longer duration of interaction with the app does not necessarily equate to meaningful engagement in the sense of adherence to the personal physical activity plan.

#### Physical Activity and Heart Rate Measurements

Participants’ physical activity and heart rate will be measured during the last 3 weeks of the study (weeks 8-10) in both study groups, using 2 wearable sensor devices (wearables). Study participants will be instructed to wear the devices daily for 3 weeks during waking hours, and to return the wearables at the final study visit (10-week follow-up). Physical activity will be measured with the ActiGraph GT9X Link accelerometer (ActiGraph LLC), worn on the nondominant wrist. Heart rate will be measured with the Polar Verity Sense device (Polar Electro Oy), worn on the nondominant upper arm or forearm. Study participants will receive both devices by post, together with written instructions and a link to an online instructional video on how to operate the wearables. Telephone support will be provided by the study team if needed.

#### Qualitative Interview

At week 10, audio-recorded semistructured interviews will be conducted in both study groups to inquire about participants’ experiences of participating in the study and any self-initiated support for physical activity during the study period. Participants in the intervention groups will also be asked about their experiences of using the aktivplan app. The interview schedule is provided in Multimedia Appendix 4.

Study participants who discontinue or deviate from the intervention protocol will be encouraged to complete all outcome assessments regardless.

### Data Collection From Health Care Professionals

#### Overview

To assess usability, user experience, and acceptance of the aktivplan app from the perspective of health care professionals, data will be collected from those members of staff at the study sites who implement the aktivplan digital health intervention with patients. These members of staff are physiotherapists or sports scientists who have provided written informed consent to have their data collected and who have received a full day of training on the aktivplan digital health intervention before the start of the study.

#### Self-Reported Questionnaires

At the beginning of the study, the health care professionals will complete the Affinity for Technology Interaction and Big Five Inventory questionnaires and rate their self-efficacy for implementing the aktivplan intervention. During the running of the study, the health care professionals will keep concurrent reflective notes on the use of the intervention, document any technical difficulties, and log their time requirements for implementing the intervention. After each aktivplan physical activity planning session, the 9-item Shared Decision Making Questionnaire [45] and items from the Nasa-Task Load Index [46] will be completed. At the end of the study, the health care professionals will again rate their self-efficacy for implementing the aktivplan intervention, and complete the mHealth App Usability Questionnaire, Mobile App Rating Scale, Unified Theory of Acceptance and Use of Technology 2, AttrakDiff mini, and the Normalization Measure Development [47] questionnaires.

#### Qualitative Focus Group

At the end of the study, a focus group discussion will elicit the health care professionals’ views on implementing the aktivplan intervention in routine clinical practice at the study sites, facilitating and hindering factors for the implementation of aktivplan, any problems experienced with the app, suggestions for improvement and new desired features of the app, and any problems and other observations regarding the conduct of the study that would need to be considered in the design of a larger definitive trial. The focus group will be audio and videorecorded and moderated by 2 researchers. The topic guide is given in Multimedia Appendix 5.

### Outcomes

The primary outcomes address the primary study aim, that is, to pilot and assess the feasibility of a future randomized controlled trial design to investigate the effectiveness of the aktivplan digital health intervention; and to assess the usability, user experience, and acceptance of the aktivplan app. The primary outcomes are presented in Textbox 3.

The secondary outcomes address the secondary study aim, that is, the collection of clinical outcomes to inform the sample size calculation for a future definitive effectiveness trial and to provide evidence toward demonstrating patient safety of the intervention. The secondary outcomes are presented in Textbox 4.

**Primary outcomes.**

- Recruitment rate (number of recruits per week, percentage of screened patients who were recruited, and percentage of eligible patients who were recruited)
- Drop-out rate (number of drop-outs at the 10-week follow-up and percentage of recruited patients who dropped out)
- Data completeness (percentage of missing data fields in study database)
- Adherence to the aktivplan digital health intervention (automated use logging of the aktivplan app and video recording of the aktivplan physical activity planning session)
- Usability of the aktivplan app (mHealth App Usability Questionnaire)
- User experience of the aktivplan app (AttrakDiff mini questionnaire)
- User acceptance of the aktivplan app (Mobile App Rating Scale and Unified Theory of Acceptance and Use of Technology 2)
- Technical reliability of the aktivplan app (number and types of technical problems and qualitative data)
- Use of alternative and additional strategies for supporting physical activity (number and types of additional strategies and qualitative data)
- Experiences and perspectives of rehabilitation professionals regarding the intervention and study procedures (qualitative data from focus groups)

**Secondary outcomes.**

- Exercise capacity (highest mechanical power output achieved during incremental cycle ergometry, peak power)
- Physical activity behavior (accelerometry and daily minutes of moderate to vigorous physical activity)
- Body mass
- Arterial blood pressure
- Blood cholesterol levels
- Glycated hemoglobin levels
- Smoking (number and type of smoking products per week)
- Health-related quality of life (EQ-5D-5L questionnaire)
- Exercise self-efficacy (Exercise Self-Efficacy Scale)
- Depression and anxiety (Patient Health Questionnaire-4)
- Kinesiophobia (selected items from the Tampa Scale of Kinesiophobia)
- Patient safety (adverse events reporting according to regulatory standards: number, type, and severity of adverse events)

### Data Management

At the study sites, data will be recorded on paper-based case report forms (CRFs). Following completion of data collection, the CRFs and electronic data collection devices (wearables, dictaphones, and video cameras) will be collated and securely stored at the study sponsor site. Data from CRFs will be entered onto a Microsoft Excel file. Quality of data entry will be assessed by independently cross-checking the accuracy of 10% of data cells. After completion of the analysis, study data will be securely archived by the sponsor for 10 years.

### Analyses

The statistical data analysis will be carried out using statistical analysis software. The analytic code will be documented and stored to ensure that the statistical analyses can be reproduced at a later date. For descriptive data analysis, the appropriate summary measures will be calculated according to data type, including measures of central tendency and spread for continuous data (arithmetic mean, SD, median, IQR, minimum, maximum, and range) and frequency, proportions, or percentages for categorical data. 95% CIs will be calculated for recruitment and drop-out rates, for adherence data, and for data completeness. Because this is a pilot study, no confirmatory inferential statistical analysis or hypothesis testing will be conducted.

An exploratory group comparison (descriptive analysis and 95% CIs) will be described for the secondary (clinical) outcome measures for both intention-to-treat and per-protocol approaches. In intention-to-treat analysis, the pattern of missing data will be assessed, but due to the small sample size no imputation of missing data will be conducted. However, we will conduct sensitivity analyses based on the minima and maxima from complete cases for each missing variable, to evaluate the potential impact of missing data in the sense of a best and worst case scenario.

The qualitative data analysis will be conducted according to the framework analysis method [48] and using qualitative data analysis software. Recordings of the patient interviews and the focus groups with health care professionals will be transcribed verbatim by a professional transcription agency. The transcripts will be checked for accuracy against the original recordings. In the reporting of qualitative findings, care will be taken to preserve the anonymity of patients and health care professionals. Relevant themes and categories from qualitative analysis will be triangulated with corresponding quantitative data to describe a comprehensive picture. Questionnaire scores for usability and acceptability of the aktivplan app will be interpreted in relation to the possible score range for each questionnaire.

## Results

Recruitment commenced on October 2, 2023. The study was completed with 34 participants recruited by May 2024, and all data were collected by August 2024. Currently, the data of the study are being analyzed and prepared for publication. The first publication of results is expected for summer 2025.

## Discussion

### Principal Findings

This feasibility study is expected to yield valuable insights to inform the design and methodology of a future definitive clinical trial evaluating the effectiveness of the aktivplan digital health intervention for regular physical activity following phase II rehabilitation. The initial implementation has already generated learnings to inform the study design. After 24 weeks, 34 (85%) participants were recruited, compared to the originally planned 40 participants, indicating that recruitment requires more time than initially anticipated. This observation supports the need for a broader, multicenter recruitment strategy in future clinical trials to ensure adequate sample size and generalizability. In addition, data on the usability, user experience, and acceptance of the aktivplan app will guide the need for further iteration before entering a definitive trial. Findings regarding the implementation in practice will inform future implementation strategies, including training for health care professionals, background technical support for health care professionals and patients, reimbursement strategies, and considerations to minimize barriers to implementation.

### Dissemination Plan

The results of this study will be published in peer-reviewed scientific journals. Due to the large volume of data generated, 3 manuscripts will report on 3 distinct aspects of the study: feasibility of the study design; usability, user experience, and acceptance of the aktivplan app; and preliminary clinical outcomes. In addition, the results will be presented at scientific conferences relevant to cardiac rehabilitation and digital technologies. A plain language summary for the cardiac community will be available on the sponsor’s website. Authorship will follow the International Committee of Medical Journal Editors authorship criteria. Professional medical writers will not be employed. It is not planned to make the participant-level dataset publicly available.

### Comparison to Prior Work

As mentioned before, the Austrian Health Interview Survey shows that only 48.1% of men and 45.1% of women aged between 18 and 64 years meet the WHO recommendation of endurance-type physical activity at moderate intensity and only 26% of men and 21.1% of women meet the WHO recommendations for both endurance-type physical activity and muscle-strengthening exercises [10]. Even after completing a cardiac rehabilitation program, 75.3% of patients in Austria fail to sustain sufficient physical activity in the long run [49]. Furthermore, as mentioned in the review of Meinhart et al [16], digital technology offers possibilities to successfully support physical activity following a cardiac rehabilitation program. Nevertheless, a recent survey by Lunz et al [50] revealed a relatively high motivation for using digital technology, but still low actual use (52%) of digital technology by health care professionals in the secondary prevention of CVD in Austria. Digital technology is mainly used for monitoring heart rate, with no established evidence-based digital health interventions for supporting physical activity habit formation [50]. Internationally, the systematic review of Luijk et al [17] describes moderate outcomes and heterogeneous quality of studies investigating the effectiveness of digital health interventions for maintaining physical activity in patients with CVD after phase II cardiac rehabilitation. However, the authors still recommend using digital health interventions in clinical practice until further evidence becomes available. Importantly, the transferability of digital health interventions between countries is often limited due to local sociocultural aspects (eg, language and representativeness of imagery) and particularities of clinical pathways and health care systems [51]. An example of the latter in Austria is the definition of 4 phases of cardiac rehabilitation, in contrast to the 3-phase model commonly used in other countries. The Austrian rehabilitation pathway offers the choice between inpatient or outpatient settings for phase II rehabilitation, and the duration and intensity of cardiac rehabilitation programs can differ to current models in other countries [3].

The aktivplan digital health intervention is therefore specifically developed for the Austrian rehabilitation pathway, and this feasibility study is deliberately conducted within the Austrian health care system to avoid variability introduced by cross-national differences in health care structures and cardiac rehabilitation programs.

### Strengths and Limitations

A strength of this study is the nature of the aktivplan intervention, which answers directly to an identified clinical need in the Austrian cardiac rehabilitation pathway, and which was developed specifically to match the cultural and systemic context in Austria using co-design methodology and patient involvement. This means that the aktivplan intervention has high clinical relevance and good sociocultural fit in the Austrian context. At the same time, this presents a limitation to the aktivplan intervention in terms of transferability to other international settings. A potential implementation of aktivplan in other countries would require an assessment of the intervention against national regulatory standards and requirements [52] and local clinical and sociocultural contexts. A further limitation to this study is the lack of blinding. Blinding of patients and health care professionals who are delivering the intervention is not possible in this case. Blinding of outcome assessors would be possible in principle; however, after early discussions with site principal investigators it was decided to conduct unblinded assessments. It was suggested that, because of the small number of available study staff and close working relationships at the study sites, blinding of assessors was not feasible, and the success of assessor blinding was unlikely. Finally, it is acknowledged that there are no prespecified criteria to judge whether, or how, to proceed with a future definitive trial [22]. Because no comparable previous studies are available for Austria, and prespecified criteria are not imposed by the funding organization, the decision whether, or how, to proceed with a future definitive trial will be made in discussion with relevant stakeholders and based on the data from this feasibility study and comparable international studies.

### Conclusions

There is a need for evidence-based interventions to support regular physical activity after discharge from phase II rehabilitation for patients with CVD or at risk of CVD. This pilot study is expected to generate valuable and comprehensive insights to inform the study design of a future definitive effectiveness trial of the aktivplan digital health intervention, guide the need for further iteration of the aktivplan app before entering a definitive trial, and inform future implementation strategies for the intervention.

## Appendix

Multimedia Appendix 1 SPIRIT checklist.

Multimedia Appendix 2 CONSORT (Consolidated Standards of Reporting Trials) checklist.

Multimedia Appendix 3 TIDieR (Template for Intervention Description and Replication) checklist.

Multimedia Appendix 4 Qualitative interview schedule.

Multimedia Appendix 5 Focus group topic guide.

## Abbreviations

BCT: behavior change technique
CONSORT: Consolidated Standards of Reporting Trials
CRF: case report form
CVD: cardiovascular disease
SPIRIT: Standard Protocol Items: Recommendations for Interventional Trials
TIDieR: Template for Intervention Description and Replication
WHO: World Health Organization

## Data Availability

Data sharing is not applicable to this paper as no datasets were generated or analyzed during this study.
