# Supplementary material for: Feasibility of the aktivplan Digital Health Intervention for Regular Physical Activity Following Phase II Rehabilitation: Protocol for a Mixed Method Randomized Controlled Pilot Study (ACTIVE-CaRe Pilot)
Source: JMIR Res Protoc. 2025 Sep 15;14:e73704. doi: 10.2196/73704 (PMC12481140; doi:10.2196/73704)
Supplement: Multimedia Appendix 5 [file resprot_v14i1e73704_app5.pdf]

Online supplement 5. Focus group topic guide (authors' translation from the original German to English).

ACTIVE-CaRe Pilot

Topic guide for the focus group with rehabilitation staff

Version 1.1
25.04.2024

www.dhp.lbg.ac.at

TABLE OF CONTENTS

1. PROJECT OVERVIEW 3
2. FOCUS GROUP 3
   2.1 Preparation and procedure 4
   2.2 Topics 5
   2.3 Topic guide 6
   2.4 Materials 9

TOPIC GUIDE FOR THE FOCUS GROUP WITH REHABILITATION STAFF

Author: Dr. Franziska Pfannerstill

Version 1.1, 25.04.2024

1. PROJECT OVERVIEW

In the **aktiv**plan project, the Ludwig Boltzmann Institute for Digital Health and Prevention in Salzburg developed an intervention in which an app supports patients and healthcare professionals in integrating heart-healthy exercise into their everyday lives.

The **aktiv**plan was developed to support healthcare professionals and patients in planning physical activity (training or exercise sessions). It can be used both as a web application and as a mobile application on a cell phone. **aktiv**plan is used by healthcare professionals together with their patients and enables the joint creation, monitoring and regular review of a personalised heart-healthy physical activity plan. Patients are involved and guided by experienced healthcare professionals in creating a personalised heart-healthy physical activity plan. Patients choose exercises and activities they enjoy and define personally meaningful goals. Through the app user interface, patients can conveniently view their plan, log activities, change activities, review their performance and access additional resources such as videos with exercise instructions. At follow-up appointments with healthcare professionals, patients can review their documented performance and discuss the plan going forward.

Healthcare professionals support the creation of personalised exercise recommendations and the ongoing monitoring and optimisation of patient performance. Healthcare staff can conveniently view patients' activity logs via the web interface. The activity logs can be exported and printed out to be filed in the medical record, they can be used as documentation for health insurance companies, and can be used at follow-up appointments for joint review and further planning with the patient.

2. FOCUS GROUP

Over the past months, a pilot study of the **aktiv**plan intervention (ACTIVE-CaRe Pilot) was conducted at two rehabilitation centres to examine its implementation, feasibility, and user experience. In order to be able to incorporate the experience gained by the healthcare professionals into the further development of the intervention, this focus group is intended to obtain their feedback. Some points are of great interest, such as the healthcare professionals' perspective on the implementation of the **aktiv**plan intervention as part of the clinical routine at the study centre, or factors that promote and hinder the use of **aktiv**plan. Also important are problems experienced, suggestions for improvement, and new desired functions for the **aktiv**plan app, as well as problems and other observations on study implementation that should be considered regarding a larger definitive trial. The focus group serves to gain qualitative insights into the **aktiv**plan intervention as part of ACTIVE-CaRe Pilot.

The focus group consists of four participants who do not necessarily know each other. They are all healthcare professionals from the rehabilitation centres that participated as study sites in ACTIVE-CaRe Pilot.

A moderator leads the focus group. The moderator is independent of the ACTIVE-CaRe Pilot study team to counteract any possible bias on the part of the participants.

Before the focus group, this guideline was drawn up, which systematically arranges the questions to ensure a stimulating yet structured discussion. The moderator leads and documents the discussion, which is tailored to the study and target group. The discussion is recorded to analyse it later and gain insights. A video camera and a voice recorder are used for this purpose, as well as notes taken during the focus group. The video recording serves as a backup recording in case of technical problems with the voice recorder, and to confirm the speaking order when transcribing the audio recording.

2.1 PREPARATION AND PROCEDURE

Before the focus group is conducted, the room is set up: The video camera and voice recorder are positioned in such a way that all participants are captured well by both devices. Paper and pens are distributed to the seats for all participants. Drinks and snacks are provided for the participants to create a relaxed atmosphere. Once the participants have taken their seats, the camera and voice recorder are turned on and the focus group begins.

The focus group is divided into four phases: The warm-up, transition, discussion and closing phases.

- **Warm-up phase:** Introduce the moderator, procedure, participants, and topic. What are the tasks and rules for engaging in a focus group, and how long does it take?
- **Transition phase:** General question about digitalisation in rehabilitation.
- **Discussion phase:** The moderator sets an approximate time frame for each question/topic and moderates the discussion.
- **Closing phase:** Finally, the moderator summarises the content discussed and asks whether there are any important topics that have not yet been dealt with/mentioned. Thanks and farewell.

2.2 TOPICS

The topics to be discussed are as follows:

A) Digital solutions in rehabilitation

B) Introduction to the **aktiv**plan intervention

- Improving the **aktiv**plan workshop for healthcare professionals

C) Implementation

- Difficulties setting up the **aktiv**plan app
- Difficulties during the consultation
- Physical activity planning with **aktiv**plan
- Experiences with person-centred goal-setting
- Advantages of **aktiv**plan with shared decision-making for physical activity planning
- Supporting patients after discharge from rehabilitation
- **aktiv**plan app notifications on behalf of the healthcare professionals
- Additional workload
- Follow-up discussions with the patients
    - a) if yes: setting/frequency
      b) which healthcare professional should conduct these
- Role management: several therapists (at the same time/one after the other)

D) Functionalities

- Missing functionalities of the app

E) Process evaluation

- Implementation as part of the clinical routine
- Improving the study planning, integration of additional tasks into everyday clinical practice
- Recommending the app to patients

F) Any other points

- Further points and experiences

2.3 TOPIC GUIDE

Section	Topic	Text
**Warm-up**	Welcome by moderator	I would like to welcome you to this focus group discussion at the end of the ACTIVE-CaRe Pilot study of the **aktiv**plan intervention. My name is XXX, and I work in XXX, and I am moderating this focus group today. My role today is to support you in conducting a constructive discussion, summarising opinions and topics, and documenting the discussion for later evaluation.<br><br>First of all, thank you once again for your cooperation and support with the study.<br>Today, I would like to ask you to review your experiences from the study and share them with us in a group discussion. You have worked directly with patients using the **aktiv**plan app and are, therefore, valuable experts for us in implementing the intervention. We are therefore particularly interested in your opinion.<br><br>I will ask you some questions that can be discussed. These are about your personal experience and opinion, so there is no "right" or "wrong". I'm interested in what worked well, and where something needs to be, or could be improved in the app, the programme of the whole intervention, the process and the implementation. You are also welcome to use your notes in your health expert logbook at any time to help you remember past situations better, or to report any problems you have encountered.<br><br>The session today will last about two hours in total, with 1½ hours for the discussion itself, and we have planned a break. The discussion will be recorded with a video camera and a voice recorder, and I will also be taking notes. The recordings and notes will later be used to evaluate the discussion. The data will be anonymised.<br><br>Do you have any questions about the process and the focus group?
**Warm-up**	Introduction of participants	I would like to ask you to briefly introduce yourself, including your name, the rehabilitation centre you work at, and what you do. You might also like to briefly summarise why you participated in the study.
**Transition**	Digital solutions in rehabilitation	Now, I would like to ask you—personally and spontaneously—what you generally think of digital solutions in rehabilitation. (e.g. which ones, for what, in which area, are positive/negative?)
**Discussion**	**aktivplan** workshop	In the further discussion, I would like to specifically discuss the individual parts of the study, from the introductory event (one-day workshop) to the implementation of the intervention.<br><br>If you think back to the **aktiv**plan workshop, how do you think this workshop for healthcare professionals could be improved?<br>- with regard to the explanation of the theoretical background to shared decision-making<br>- with regard to the use of **aktiv**plan<br>- with regard to the explanation of ACTIVE-CaRe Pilot study procedures<br>(Possible follow up: What should you have been better prepared for in the introductory event? Materials, specific content, duration?)

**Discussion**	Difficulties setting up	Please remember your very first **aktiv**plan physical activity planning session with patients.<br>Did you have any difficulties setting up the **aktiv**plan app?<br>If so, how were these resolved?
**Discussion**	Difficulties conversation	What difficulties did you encounter leading the conversation during the session?<br>How did you deal with it?
**Discussion**	Personalised goal-setting	How did you experience the personalised goal-setting for the time after rehabilitation during the session?
**Discussion**	Physical activity planning	What did you do differently than usual when devising the physical activity plan with the **aktiv**plan website and app**?**
**Discussion**	Advantages of shared decision-making	Do you see any advantage in the focus on shared decision-making in **aktiv**plan when devising the physical activity plan?<br>If so, what is it?
**Discussion**	Monitoring patients after rehabilitation	If you now think about the time when patients have been discharged from rehabilitation:<br>How did you experience the monitoring of your patients with the **aktiv**plan app after discharge from rehabilitation?
**Discussion**	**aktiv**plan notifications	Patients receive automated notifications on your behalf in the **aktiv**plan app.<br>How do you feel about this?<br>How do you feel about your name being shown as the sender used in these automated notifications?
**Discussion**	Follow-up discussion	Did you feel that there should have been a planned follow-up session with the patients during the current study (10-week follow-up period)?<br><br>Imagine that the **aktiv**plan intervention is now being rolled out across the board at your centre and patients are expected to use it for a whole year: In your opinion, should planned follow-up sessions with patients take place?<br>a) If so, in which setting and at what frequency?<br>b) In your opinion, which healthcare professional(s) should lead these sessions?
**Discussion**	Additional workload	How did you experience the additional workload during the entire time the study was being carried out?<br>(ONLY in relation to the **aktiv**plan intervention, not in relation to study-related procedures such as data collection with questionnaires).
**Discussion**	Role management	Imagine that the **aktiv**plan intervention is now to be rolled out in clinical practice at your rehabilitation centre:<br>In which situations do you think it would be necessary that another colleague has access to your patients in **aktiv**plan?<br>How did you manage periods of absence during the study, e.g., when you were on vacation or off sick?
**Break**		Break

**Discussion**	**aktiv**plan content	Now please think about the content of the **aktiv**plan website and app:<br>- Information provided in the physical activity planning guide<br>- Stored exercise descriptions and videos<br><br>Are these sufficient / too extensive / too few?<br>Is content missing - if so, what is missing?
**Discussion**	**aktiv**plan functionality	Now please think about the functionalities of the **aktiv**plan website and app:<br>- Physical activity planning guide<br>- Calendar function<br>- Physical activity planning<br>- Personalised goal-setting<br>- Automated messages to patients<br>- Personalised messages to patients<br>- Status input of the patients<br><br>What functionality do you think is missing in **aktiv**plan?<br>Did something not work according to your expectations?
**Discussion**	Implementation in clinical practice	Now I would like to ask you a few more questions about bringing the **aktiv**plan intervention and the ACTIVE-CaRe Pilot study to your rehabilitation centre:<br>How did you experience the implementation of the **aktiv**plan intervention as part of clinical practice at your rehabilitation centre?
**Discussion**	Improving integration into clinical routines	From your point of view, how could the ACTIVE-CaRe Pilot study planning be improved to integrate the additional tasks that arise for you as healthcare professionals into your clinical routines in the most convenient way?
**Discussion**	**aktiv**plan recommendation	Would you personally recommend the **aktiv**plan app to patients?<br>If yes - why?<br>If not - why not?
**Closing**	Further comments	Finally, we have allowed some time for comments that have not yet been covered in the discussion:<br>Are there any other points you would like to say or discuss, or other experiences from the last months that you would like to share with us?
**Closing**	Thanks and farwell	Thank you very much for taking the time to take part in this discussion and for supporting the study so actively. The results of the discussion will be summarised anonymously.<br>Your feedback is valuable to us to improve the **aktiv**plan intervention and the planning of the follow-up study to ACTIVE-CaRe Pilot.<br><br>We would also like to ask you for your records (document "Records of the rehabilitation staff") so that we can also use your questionnaires and notes for further evaluation.<br><br>We would be very happy to continue working with you, thank you again.

2.4 MATERIALS

Video camera	☐
Voice recorder	☐
Pens and paper for participants' notes	☐
Material for notes/transcript of the co-moderator	☐
Records of the rehabilitation staff (to be brought along by the healthcare professionals, then handed over by the moderation team to the study management at the Ludwig Boltzmann Institute)	☐
